# A framework for uniting space and time in the mind and brain

**DOI:** 10.3389/fcogn.2025.1611855

**Published:** 2025-10-07

**Authors:** Troy M. Houser

**Affiliations:** Department of Psychology, University of Oregon, Eugene, OR, United States

**Keywords:** temporal discounting, successor representation, exploration-exploitation, cognitive representation, decision-making

## Abstract

Kant argued that all experience is perceived through the lens of *a priori* concepts of space and time. That is, Kantian philosophy supposes that knowledge is formatted in terms of space and time. This article argues that space can be reduced to time and thus that the only *a priori* concept used to format knowledge is time. To build this framework, the article focuses on how humans discount time when making intertemporal choices. Celebrated temporal discounting models, such as the exponential and hyperbolic discounting models, are reviewed before arguing in favor of a more ecologically motivated account that suggests that hyperbolic discounting emerges from exponential discounting and uncertainty. The ecological account of temporal discounting is then applied to spatial navigation. Along the way, findings from neurobiology and principles from computational mechanisms are used to substantiate claims. Reducing space to time has important implications in cognitive science and philosophy and can inform a suite of seemingly distinct literatures.

## Introduction

Experience occurs in both space and time. Thus, everything we do is imbued with a spatiotemporal aspect, yet, as Aristotle remarked many years ago, our mental lives lack an objectively spatial component but seem to abide by the same temporal dynamics as the physical world. Therefore, either space is an innate concept that animals are aware of and understand prior to experience or the idea of space is derived from temporal experience. In this article, I argue for the latter: the view that space is derived from time assumes that space is a latent construct that has to be inferred by the brain, given its lack of direct access to the world. Although the brain does not have direct access to the spatial information in our environment, it has to operate in time, and thus, I assume that the brain does have access to temporal information. This perspective is inspired by philosophical frameworks, such as that espoused by Henri Bergson, Plato's notion of time discussed in *Timaeus*, and Kant's *a priori forms of intuition*, although this article focuses on a neurobiological account.

In what follows, I use research on intertemporal decision-making to elucidate how humans represent time in the mind and brain and how such representations shape decision-making. Specifically, I briefly review a number of computational models that capture intertemporal choices, including the canonical exponential and hyperbolic discounting models, as well as more recently proposed Bayesian accounts of temporal discounting behavior. I then evaluate temporal discounting from an ecological perspective and provide an explanation for how hyperbolic discounting emerges from exponential discounting with heterogeneous discounting rates, an idea originally introduced by (Sozou 1998). This idea leads to a plausible neurobiological account of temporal discounting. Assuming that psychological time is represented by a hypothetical neuron population that discounts time exponentially but with heterogeneity, then this neuron population could also give rise to spatial representations. To explore this possibility, I next review how spatial representations can be derived from the successor representation (SR; Dayan, 1993), which has substantial computational and neurobiological support. Finally, I illustrate the equivalence between a multiscale SR and heterogeneous temporal discounting. I conclude with some potential applications of the proposed framework and testable predictions that follow logically from the guiding principles laid out.

## Time

To see how the brain derives its concept of space from its experience with time, I first review how the brain represents time. There are a few prominent theories for how the brain tracks time, including the population and rate coding approaches (MacDonald et al., 2011; Pastalkova et al., 2008). However, here I specifically focus on temporal *representation*. That is, for example, while a single cell's firing rate may track clock time, it is known that consciously accessible representations of time are anything but perfect. Therefore, I assume that such a cell's firing rate is correlated with, but does not *represent*, time. Notably, in this article, no claims regarding the objective nature of time or space are made, although, in the discussion, I speculate on how and why the framework proposed is (partially) consistent with Kant's philosophy. For representing time, I turn to cognitive science. The intertemporal choice task, in particular, is an excellent candidate paradigm for studying representations of time.

### Background on intertemporal choice

Intertemporal choices are trade-offs between gains and losses occurring at different times (Frederick et al., 2003; Loewenstein and Thaler, 1989). For example, one might have to decide between spending money on a night out that would be immediately rewarding or saving the money to go on an overseas holiday sometime in the future. Decision-making involving outcomes over time is ubiquitous. It characterizes many important life decisions, such as choosing a life partner, saving money for retirement, changing diets for future health, and conserving energy for an upcoming arduous task. Why does everyone not choose health, to save money, and to conserve energy? As was noted in early studies, the only reason for not choosing health, money, and energy is because its payout occurs later. The effect that delaying gratification has on behavior was studied in Hull's (1932) *goal gradients* and the famous Stanford marshmallow experiment (Mischel and Mischel, 1983). These early experiments noted that later rewards are *discounted* (i.e., lessened or reduced in effect). Moreover, they produced considerable research into *how* rewards are discounted with time.

The first formal treatment of temporal discounting emerged from a generalized version of Herrnstein's (1961) matching law, which says that animals allocate their behavior *B* between two choices, 1 and 2, in proportion to their rates of reinforcement *R*:


(1) $$\begin{array}{l} \frac{B_{1}}{B_{2}}=\frac{R_{1}}{R_{2}}. \end{array}$$

If we imagine that the behaviors are temporal choices *T*, then we have the matching law of intertemporal choice:


(2) $$\begin{array}{l} \frac{T_{1}}{T_{2}}=\frac{R_{1}}{R_{2}}. \end{array}$$

(Baum 1974) generalized the simple matching law to evaluate intertemporal choice:


(3) $$\begin{array}{l} \frac{T_{1}}{T_{2}}=b{\left(\frac{R_{1}}{R_{2}}\right)}^{s}, \end{array}$$

where *b* is a bias parameter or a value meant to capture influences beyond reinforcement and *s* stands for sensitivity to reinforcement. Say one is offered $10 in 5 days or $20 in 10 days. If we plug in these numbers, *T*_1_ = 5, *T*_2_ = 10, *R*_1_ = 10, and *R*_2_ = 20. This makes the left-hand side of the equation 0.5. When *s* = 1, Equation 3 reduces to Equation 2, and one is simply biased toward one or the other alternative (${\left[\frac{R_{1}}{R_{2}}\right]}^{1}=\left[\frac{R_{1}}{R_{2}}\right]$). When 0 < *s* < 1, the left-hand side “undermatches” $\frac{R_{1}}{R_{2}}$ (e.g., using the previous example, when *s* = 0.5 and *b* = 1, the right-hand side of Equation 3 is 0.71), meaning that not enough behavior is allocated to the immediate reward. When *s*>1, the left-hand side “overmatches,” meaning not enough behavior is allocated to the later reward. While the matching law of temporal discounting does account for individual sensitivity to reward ratios, it lacked a formulation of subjective value, which is important because experience tells us that preferences are subjective (Peters and Büchel, 2010). Some people are more patient than others (Bornovalova et al., 2009; Daruna and Barnes, 1993; Jauregi et al., 2018), and some people take risks, while others play it safe (Bornovalova et al., 2009; Lejuez et al., 2004; Zuckerman and Kuhlman, 2000). Moreover, the matching law does not assume that animals discount rewards at all, which flies in the face of numerous experiments showing that animals consistently favor immediate gratification even when the reinforcement rates for both options are equal (Rachlin, 2006). Behavioral economic models provided a solution to these problems.

Given that matching is a strategy for reward maximization (Rachlin, 2006; Rachlin et al., 1976), it is also common to view temporal discounting as a utility function. Indeed, Samuelson's (1937) model of discounted utility was the standard model of intertemporal choice for a while. It suggests that future rewards are discounted exponentially with time:


(4) $$\begin{array}{l} V_{LL}=R_{LL}\exp\left(-kD\right), \end{array}$$

where *V*_*LL*_ is the subjective value of the larger-later reward, *R*_*LL*_ is the larger-later reward offered, *D* is the time delay until payout, and *k* is called the temporal discount factor. If *k* = 0, there is no discounting and *V*_*LL*_ = *R*_*LL*_. As *k* increases, however, the multiplier that scales *R*_*LL*_, *exp*(−*kD*), shrinks and thereby also shrinks the subjective value of the larger-later reward. For example, assuming *R*_*LL*_ = *$*10 and *D* = 5 days, *V*_*LL*_ = *$*9.51, *$*7.79, and *$*6.07 when *k* = 0.01, 0.05, and 0.10, respectively. Thus, *k* is free to vary. The *k* parameter became associated with individual levels of patience, or impulse control (Ainslie, 2022), for the smaller *k* is the more likely one is to wait for the larger-later reward rather than opting for the immediate gratification. Consistent with the centuries-old theory of expected utility, the exponential model of temporal discounting assumes that people's preferences are rational. That is, if one prefers $100 in a year over $110 in 2 years, they will also prefer $100 today over $110 in a year. This is rational because the delay and reward ratios are the same in both scenarios, meaning that there is nothing new about the second scenario that should change their preference. Of course, Kahneman and Tversky's (1979) explosive prospect theory put an end to belief in humanity's perfect rationality. For example, it is not uncommon for one to prefer $110 in 2 years over $100 in a year but also $100 today over $110 in a year. These preference reversals can be modeled if rewards are discounted hyperbolically with time. (Ainslie 1975) noted that the matching law applied to a single delayed reward would yield a hyperbolic discount curve:


(5) $$\begin{array}{l} V_{LL}\propto \frac{R_{LL}}{D}. \end{array}$$

The standard formula for hyperbolic discounting became (Mazur, 1987)


(6) $$\begin{array}{l} V_{LL}=\frac{R_{LL}}{1+kD}. \end{array}$$

**Figure 1:**
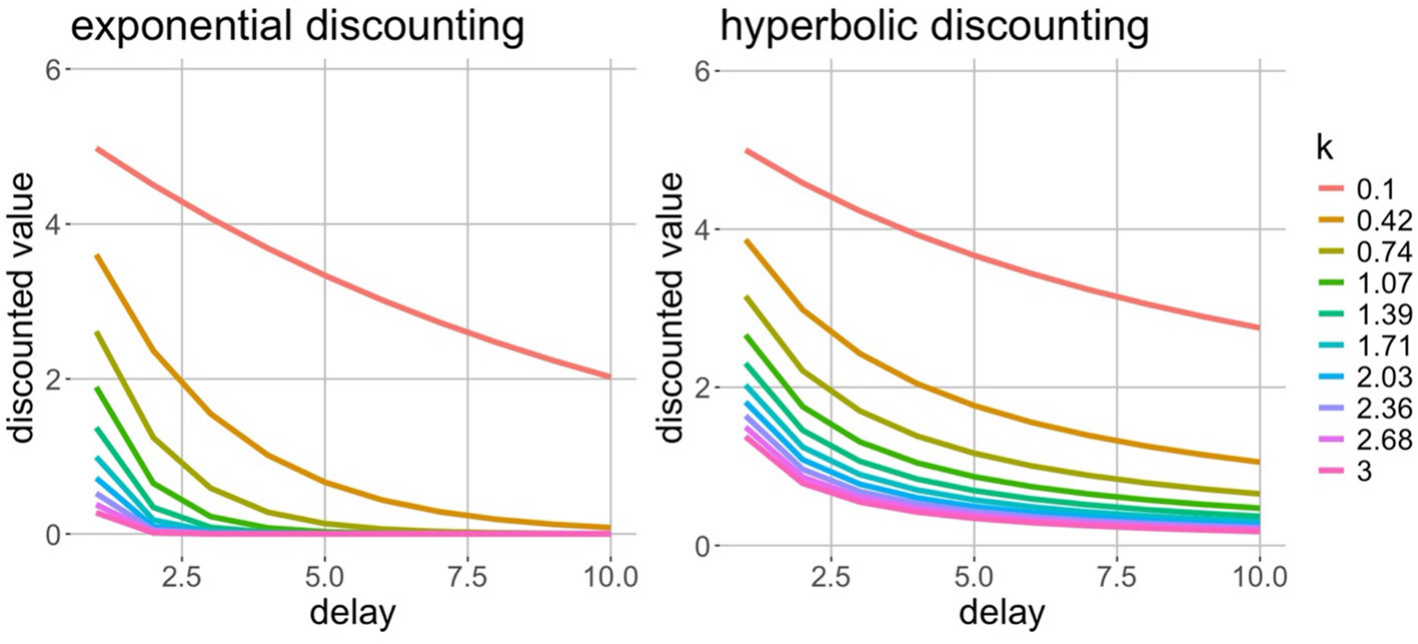
Temporal discounting rates. Both plots show a range of temporal delays on the *x*-axis and hypothetical discounted values of the delayed rewards on the *y*-axis. Color represents the rate of temporal discounting. On the left, the relationship between discounted value and delay is exponential, and on the right, the relationship is hyperbolic. It can be seen that hyperbolic discounting suggests a more gradual decline in value with delay.

Hyperbolic discount curves decrease more sharply for smaller delays than exponential discount curves (i.e., hyperbolic curves have greater concavity), which was seen as crucial for explaining seemingly irrational addictive behaviors (Ainslie and Monterosso, 2003; Monterosso and Ainslie, 2007). The marked success of exponential and hyperbolic temporal discounting models (Figure 1) at capturing a suite of mental health disorders has led to it being proposed as a transdiagnostic marker of psychopathology (Lempert et al., 2019). Moreover, recent studies have demonstrated the global generalizability of temporal discounting (Rieger et al., 2021; Ruggeri et al., 2022, 2023; Wang et al., 2016). While informative and useful for prediction, deciphering what these computational models mean for our representations of time is not easy.

### Ecologically rational intertemporal choice

When faced with two options that do not have an obvious winner, people often deliberate considerably, thinking through possible payoffs and consequences and reflecting on their own goals or histories. This process forces a cognitive agent to construct and search through a mental space of options and outcomes (Bulley and Schacter, 2020). This highly executive process was initially posited to be underpinned by activity in the prefrontal cortex (McClure et al., 2004, 2007) and is contrasted with a separate, impulsive neural substrate in the limbic system, which was suggested to explain the common finding that animals are biased to choose immediate gratification (Ainslie, 2022; McClure et al., 2004, 2007; Peters and Büchel, 2011; Ruggeri et al., 2022). Evidence indeed supports the involvement of the prefrontal cortex in temporal discounting (Ciaramelli et al., 2021; Kable and Glimcher, 2007; Koban et al., 2023; Peters and Büchel, 2010, 2011). Along similar lines, hippocampal damage in rats shows increased temporal discounting (Cheung and Cardinal, 2005; Mariano et al., 2009; Rawlins, 1985; Rawlins et al., 1985), which is particularly intriguing because hippocampal damage also impairs people's ability to imagine the future (Hassabis et al., 2007). Based on these findings, it has been suggested that the hippocampus contributes to decision-making by projecting oneself into the future (Bar, 2009; Schacter et al., 2007, 2012; Schacter and Addis, 2007). The hippocampus is also known to construct cognitive maps of one's spatial environment using place cells, which are neurons that fire when an animal occupies a specific spatial location (O'Keefe and Dostrovsky, 1971), making the hippocampus a prime candidate for investigating the inference of space from time.

In one of the first studies on the relationship between episodic future thinking and temporal discounting, participants were administered a classical intertemporal choice task involving a series of choices between smaller-sooner and larger-later rewards (Peters and Büchel, 2010). Importantly, the authors introduced a novel condition featuring presentation of episodic cue words referring to real, subject-specific events planned for the day of reward delivery in the intertemporal choice task. It was reasoned that such cues would induce more episodic future thinking and reduce temporal discounting. This is precisely what was found. It was also found that the episodic cue condition induced stronger functional connectivity between the prefrontal cortex and hippocampus (Peters and Büchel, 2010). Thus, it may be the case that the prefrontal cortex draws on mental simulations of the future by the hippocampus to make intertemporal decisions. This neural mechanism is considered evolutionarily advantageous (Boyer, 2008), as it biases choice toward what is better in the long run (Benoit et al., 2011).

Interestingly, this link between temporal discounting and mental simulation has also been used to explain the general bias toward immediate gratification. Given that episodic future thinking decreases temporal discounting and consumption of larger-later rewards is better, we might ask why people do not always employ episodic future thinking strategies to make intertemporal choices. First, episodic future thinking is effortful (Bulley and Schacter, 2020). Thus, to mentally simulate a future reward, one must expend cognitive resources. The more precisely one imagines the future, the more resources that are required (Gershman and Bhui, 2020). As such, a trade-off exists within the trade-off between choices, which is between how much attention to pay toward simulating the future and the magnitude of the future reward. Building on a Bayesian model of hyperbolic discounting (Gabaix and Laibson, 2017), (Gershman and Bhui 2020) modeled the magnitude effect of temporal discounting—the finding that people discount time less if the magnitude of the future reward is relatively large—using a model that estimates temporal discounting on a trial-by-trial basis. This model assumes that the mentally simulated future reward is a randomly drawn sample from a Gaussian distribution with mean *R*_*LL*_ and variance $\sigma_{R}^{2}$, $s\sim N\left(R_{LL},\sigma_{R}^{2}D\right)$. Note that the uncertainty associated with the mental simulation scales with the magnitude of the delay. The key term in this model is the mental simulation noise $\sigma_{R}^{2}$, which can be approximated as the variance of all encountered rewards $\sigma_{r}^{2}$. This approximation makes implicit that one's prior uncertainty is proportional to the variability of experienced rewards so far. This also captures possible order effects in the intertemporal choice task. Order effects in intertemporal decision-making are rarely considered because *k* is assumed to be stable across tasks. To capture the magnitude effect (i.e., bring temporal discounting under adaptive control), mental simulation noise $\sigma_{R}^{2}$ becomes $\frac{\sigma_{r}^{2}}{\beta |R|}$, where β, or reward sensitivity, scales the magnitude of the offered rewards. With increasing reward sensitivity, the simulation noise shrinks, which will, in turn, minimize the variability in the sample *s*. In this model, the discount rate is estimated on each trial as


(7) $$\begin{array}{l} k=\frac{\sigma_{R}^{2}}{\sigma_{r}^{2}}, \end{array}$$

which gets plugged into Equation 6, meaning that the discount rate is the ratio of mental simulation noise to observed noise. If mental simulation noise is large compared to observed noise, the discount rate increases. If mental simulation noise is small compared to observed noise, the discount rate decreases, facilitating more patience. Together, temporal discounting is a multifaceted phenomenon shaped by individual differences, as well as reward and uncertainty. However, how more uncertainty biases individuals toward immediate gratification remains unclear. Better yet, *why* does this occur?

One account suggests that premature commitment to suboptimal choices is an adaptive compensatory strategy for high levels of uncertainty that could cause indecision (Salvador et al., 2022). For example, if one has to choose between $10 now and a draw from a range of possibilities, one might waste precious time trying to reduce the uncertainty of the second option. Consistent with this notion, evolutionary theories have posited the possibility that impulsivity can be adaptive (Stevens and Stephens, 2010). Intertemporal choice is intimately bound up with foraging (Houser, 2024; Namboodiri et al., 2016; Stevens and Stephens, 2010). An animal may wander upon some food, at which point they exploit this resource; however, each bite depletes the resource, and the environment may or may not be changing. At what point does the animal switch to exploring for more resources? This so-called exploration–exploitation dilemma *is* an intertemporal choice: exploiting a known resource is analogous to immediate gratification, while exploring incurs a temporal cost but with the hope that a larger reward can be found. Furthermore, exploration is associated with more uncertainty, as is the larger-later reward in intertemporal decision-making. A large reward may exist somewhere in the environment, but it also might not. (Stevens and Krebs 1986) showed that when travel times between resources (i.e., larger-later reward delay) are long, exploiting longer travel times is better than short travel times. Environments with plenty of resources will have shorter travel times. Thus, from the perspective of someone in a plentiful environment, overexploitation may appear impulsive, yet overexploitation is adaptive in environments where resources are few and far between (Fenneman and Frankenhuis, 2020). This is fascinating because it means that overexploitation can be a decision-making strategy that depends on thinking about the future, a process not often linked to impulsivity. Together, it is plausible that the so-called present bias in temporal discounting is a function of the risk that the larger-later reward will not be available in the future.

Take, for example, some animal that caches food for the winter. Then, the animal notices a predator lurking in the environment, so the animal digs up its stash and gobbles up the food. On the surface, this behavior might appear myopic; however, it is a rational response to the realization that the predator may steal its stash before winter ends (Houston et al., 1982). If the reward is a future reproductive opportunity, the animal may die before the reproductive act (Iwasa et al., 1984). For humans, an investment opportunity comes with the risk that the stock market collapses. It is thus convenient to define a survival function by specifying the likelihood that a future reward can be realized following some temporal delay, whose inverse can be interpreted as the risk of a reward not being realized. For each unit of time, such risk is known as the hazard rate *H*:


(8) $$\begin{array}{l} H\left(D\right)=\frac{1dS}{SdD}, \end{array}$$

where *S* is the survival function. If the hazard rate is constant across all temporal delays, the survival function equals the exponential discounting model (Sozou, 1998). I already mentioned how animals exhibit preference reversals consistent with hyperbolic discounting, so why should the hazard rate, which is a more ecologically motivated framework, drop off in such a way? (Sozou 1998) proposed that animals do assume constant hazard rates, but that uncertainty lies in what the rate is. For simplicity, I assume that the hazard rate can take one of *n* discrete values, such that the survival function is


(9) $$\begin{array}{l} S\left(D\right)=p_{1}\exp\left(-{\text{λ}}_{1}D\right)+p_{2}\exp\left(-{\text{λ}}_{2}D\right)+\ldots +p_{n}\exp\left(-{\text{λ}}_{n}D\right), \end{array}$$

where *p*_*i*_ is the probability associated with hazard rate λ_*i*_. This is the prior distribution of hazard rates. Generalizing, the Laplace transform of this distribution yields a solution to the case when the hazard rate is continuous:


(10) $$\begin{array}{l} S\left(D\right)=\int_{0}^{\infty }f\left(\text{λ}\right)\exp\left(-kD\right)d\text{λ}, \end{array}$$

and by setting the prior probability density function *f*(λ) to $\frac{1}{k}exp\left(-\;\frac{\text{λ}}{k}\right)$,


(11) $$\begin{array}{l} S\left(D\right)=\frac{1}{1+kD}. \end{array}$$

**Figure 2:**
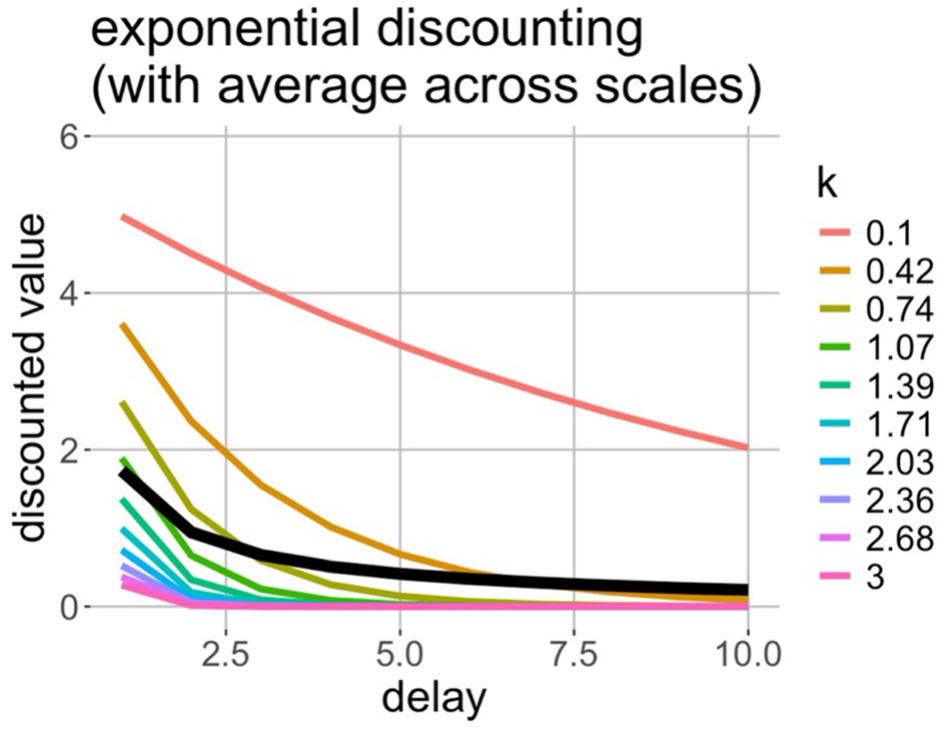
Averaging across exponential discounting at different rates results in a hyperbolic relationship between discounted value and delay. The *x*-axis denotes the temporal delay until payout, and the *y*-axis denotes the hypothetical discounted value of the reward to be received in the future. Each colored curve represents an exponentially discounted reward at a different rate. The black curve is the average of the colored curves. It can be seen that the average across exponential curves resembles a hyperbolic curve.

The remarkable result of this formulation is that taking the Laplace transform of the prior distribution of hazard rates, each one of which individually is consistent with exponential discounting, yields a hyperbolic discounting curve. This is approximately the same as averaging across exponential discount functions, as visualized in Figure 2. In the brain, we might imagine a population of discounting cells, each of which is tuned to a different hazard rate, that collectively produce hyperbolic discounting. Cell types that perform similar functions have been observed in the entorhinal cortex (Bright et al., 2020) and hippocampus (Cao et al., 2021; Howard and Eichenbaum, 2015; Salz et al., 2016). Can this hypothetical population of neurons also give rise to our concept of space?

## Space

The hippocampus contains fascinating neurons, called place cells, that fire when an animal occupies a particular spatial location (O'Keefe and Dostrovsky, 1971). Place cells have been hypothesized to serve as the neural substrate for cognitive maps (O'keefe and Nadel, 1979), and a wealth of empirical evidence supports this notion (Behrens et al., 2018; Bellmund et al., 2018). Experimental work on cognitive maps initially focused on how the brain represents simple environments, such as an open arena (Wilson and McNaughton, 1994). More recently, complex environments resembling many natural environments that are highly structured have been employed (Zhang et al., 2024). Some have suggested that what place cells represent is more akin to a cognitive graph than a cognitive map (Burgess and O'Keefe, 1996; Kumaran and Maguire, 2005; Muller et al., 1996; Peer et al., 2021), the difference being that cognitive graphs feature discrete locations as nodes and distance between nodes equals the number of edges separating the nodes, whereas cognitive maps utilize Euclidean space. The truth is likely some combination of the two (Peer et al., 2021); however, I believe cognitive graphs make the following discussion more digestible.

If we imagine that space can be represented with nodes and edges (e.g., the Empire State Building and Madison Square Garden can be denoted as two nodes with four edges, or city blocks, between them), then *anything* can be represented spatially. That is, we can just as easily imagine two nodes being red and green separated by three edges (assuming that yellow and orange nodes are in between the red and green nodes). This allows us to talk about color in spatial terms. Indeed, research has elucidated neural underpinnings for odor (Bao et al., 2019), auditory (Aronov et al., 2017), visual (Nau et al., 2018), social (Park et al., 2021), emotion (Qasim et al., 2023), reward (Nitsch et al., 2023), semantic (Viganò et al., 2021), and virtual (Bellmund et al., 2016; Doeller et al., 2010; Julian and Doeller, 2021) spaces, all residing within the entorhinal-hippocampal circuitry. Similarly, conceptual information, which is known to be organized mentally along metric spaces of physical similarity (Jäkel et al., 2008; Kruschke, 1992; Love et al., 2004; Nosofsky, 1986, 1987; Nosofsky and Kruschke, 1992), is also represented in the hippocampus (Bowman et al., 2020; Bowman and Zeithamova, 2018) and entorhinal cortex (Constantinescu et al., 2016). Moreover, navigation in abstract mental spaces recapitulates many characteristics of spatial navigation (Behrens et al., 2018; Bellmund et al., 2018; Giron et al., 2022; Hills et al., 2008, 2013; Meder et al., 2021; Schulz et al., 2018, 2019; Wu et al., 2018, 2020, 2021). The hippocampus representing abstract spaces is also aligned with Eichenbaum's (2017a,b) theory that cognitive maps of one's environment are merely a special case of the hippocampus's much broader role in relational memory (Dusek and Eichenbaum, 1997). Relational memory, however, does not explain hippocampal involvement in episodic future thought, planning, or intertemporal choice.

An emerging literature attempting to unite the numerous findings regarding cognitive maps has approached the topic from a reinforcement learning perspective. Reinforcement learning is primarily concerned with the problem of reward maximization (Sutton and Barto, 1998). This is a useful lens through which to evaluate the hippocampal function of spatiotemporal representations because reward maximization in the long run requires an ability to both predict the future and represent decision environments (Gershman, 2018). These two requirements of successful reinforcement learning represent the more abstract computational necessities of efficiency and flexibility. Predicting the future allows one to make efficient decisions (e.g., if I know I have a doctor's appointment in the afternoon, I can prioritize small assignments at work because I will not have the time to complete a large assignment). Representing the environment allows one to be flexible (e.g., a cognitive map of the city can facilitate shortcuts or detours if a road closes). A recently revived idea in reinforcement learning, known as the SR (Dayan, 1993), combines both efficiency and flexibility by constructing a predictive map.

The SR learns a predictive map like how the celebrated temporal difference algorithm (Sutton and Barto, 1998) learns a value function. The crucial difference is that SR learning is vector-valued; that is, it iteratively updates values of an entire vector rather than a single point. This vector represents the likelihood of transitioning to any other state, given a starting state. This allows the SR to learn long-term relationships and transition probabilities between states or locations. Specifically, the SR can be constructed via a count-based temporal difference update (Momennejad, 2020):


(12) $$\begin{array}{l} M\left(s_{t}\right)=M\left(s_{t}\right)+\alpha \left(I\left[s_{t}=s^{\prime }\right]+\gamma M\left(s^{\prime }\right)-M\left(s_{t}\right)\right). \end{array}$$

Here, *M* is the SR, *I*[·] is an indicator function where every element is 0 except if the argument is true, *s*_*t*_ is the current state, *s*′ is the successor state, α is the learning rate, and γ is the temporal discounting rate. Importantly, *M* is a matrix with rows and columns equal to the number of states in the environment. Each *ij*th entry in the matrix represents a transition from state *i* to state *j*. Imagine being at home and that you frequent the Empire State Building and Madison Square Garden. *M* is a 3 × 3 matrix representing transitions between each of these locations (home, Empire State Building, Madison Square Garden). Say you are home and have plans to see a comedy show at Madison Square Garden. *S*′ represents Madison Square Garden and *S*_*t*_ represents home, making $M\left(s^{\prime }\right)-M\left(s_{t}\right)=\left[0,0,0\right]-\left[0,0,0\right]=\left[0,0,0\right]$. When we add the indicator function, we get [0, 0, 1]+[0, 0, 0] = [0, 0, 1]. The resulting vector, [0, 0, 1], denotes the fact that you are going to Madison Square Garden. Adding this transition to the original “home vector,” *M*(*s*_*t*_), yields the vector [0, 0, 1], which in the row of *M* corresponding to home now means that when at home, you are most likely to go to Madison Square Garden. After numerous transitions, a predictive map is gradually built up that represents the likelihood of all possible transitions between home, Empire State Building, and Madison Square Garden.

By learning transitions among states, the SR can obtain knowledge of space without encoding anything inherently spatial. Say I want to encode the layout of my living room, which is essentially an open arena. The location one step to my right has to be represented in this cognitive map as one step to my right, the location one step to my left has to be represented as one step to my left, and so on to be able to move about in my living room. Instead of trying to *a priori* figure out what space is, my brain can simply record my transition statistics: I am much more likely to take one than two, three, or four steps to my left, just as I am much more likely to take one than two, three, or four steps to my right. Thus, when in my current location, the SR would infer the likelihood of my next move being one step away. And once I took one step, the next likely move would be to take another single step from that location. The idea of a SR in the mind and brain thus predicts that representing successor states contains implicit knowledge of space without needing to encode anything inherently spatial.

**Figure 3:**
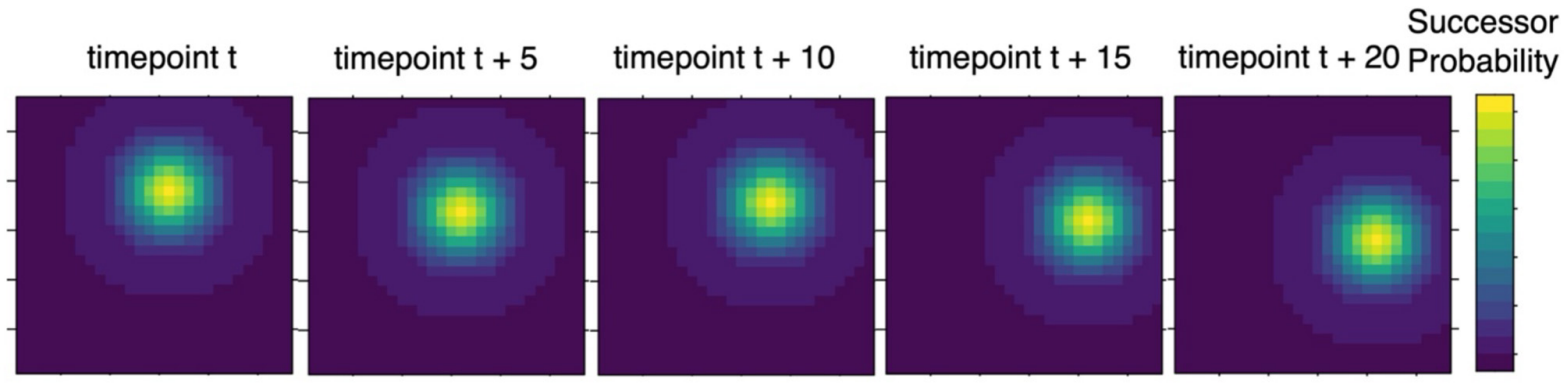
Example of place cell activity across time. Each grid in the plots can be construed as a matrix of place cell activities, such that each matrix entry represents a single place cell's activation level. Assuming that the location of the animal is at the entry that is brightest, place cell activity decays with distance from this location, resulting in a 2-dimensional multivariate Gaussian distribution (i.e., a radial basis set function). Traditionally, this activation pattern has been interpreted as the tuning of each place cell to space; however, an alternative account is that it represents the likelihood of transitions. That is, one's next step is likely to be to an adjacent spatial location. Thus, place cell activation may denote successor probability.

Intriguingly, SRs explain hippocampal place cell activity (Gershman, 2018; Stachenfeld et al., 2017). Place cells have receptive fields that resemble Gaussian tuning curves. That is to say that a place cell is most active in a given location, and its activity decreases as a function of a two-dimensional Gaussian distribution, which is precisely the activation pattern ascribed to place cells by the SR (Figure 3). A two-dimensional Gaussian tuning curve means that a place cell will fire maximally to adjacent locations. Traditional models of place cell activity suggest that this is because place cells are receptive to a patch of space that can be modeled as a two-dimensional Gaussian, whereas the SR suggests that this is because nearby locations are the likeliest successor states. Evidence for predictive maps in the hippocampus has been observed (Ekman et al., 2023; Garvert et al., 2017, 2023; Gershman, 2018; Momennejad, 2020; Sosa and Giocomo, 2021; Stachenfeld et al., 2017).

Predictive maps also lend themselves nicely to the phenomenon of hippocampal replay (Skaggs and McNaughton, 1996), which is when place cells reactivate in the order that they activated previously when foraging at a temporally compressed rate (Foster, 2017; Liu et al., 2019; Wittkuhn et al., 2021). Substantial work shows that hippocampal replay contributes to memory consolidation of salient traveled paths (Deuker et al., 2013; Gagne et al., 2018; Liu et al., 2019; Mattar and Daw, 2018; Momennejad, 2020; Wittkuhn et al., 2021). By encoding multistep predictive maps, the SR can retrace a sequence of transitions that led to reward. This also means that the SR can, in a sense, unroll a sequence of most likely transitions to simulate a future trajectory (Wittkuhn et al., 2021). This latter notion has been termed “preplay” (Buhry et al., 2011; Dragoi and Tonegawa, 2013), and it is involved in planning and prospection (Corneil and Gerstner, 2015; Mattar and Daw, 2018; Ólafsdóttir et al., 2018).

## Uniting space and time

**Figure 4:**
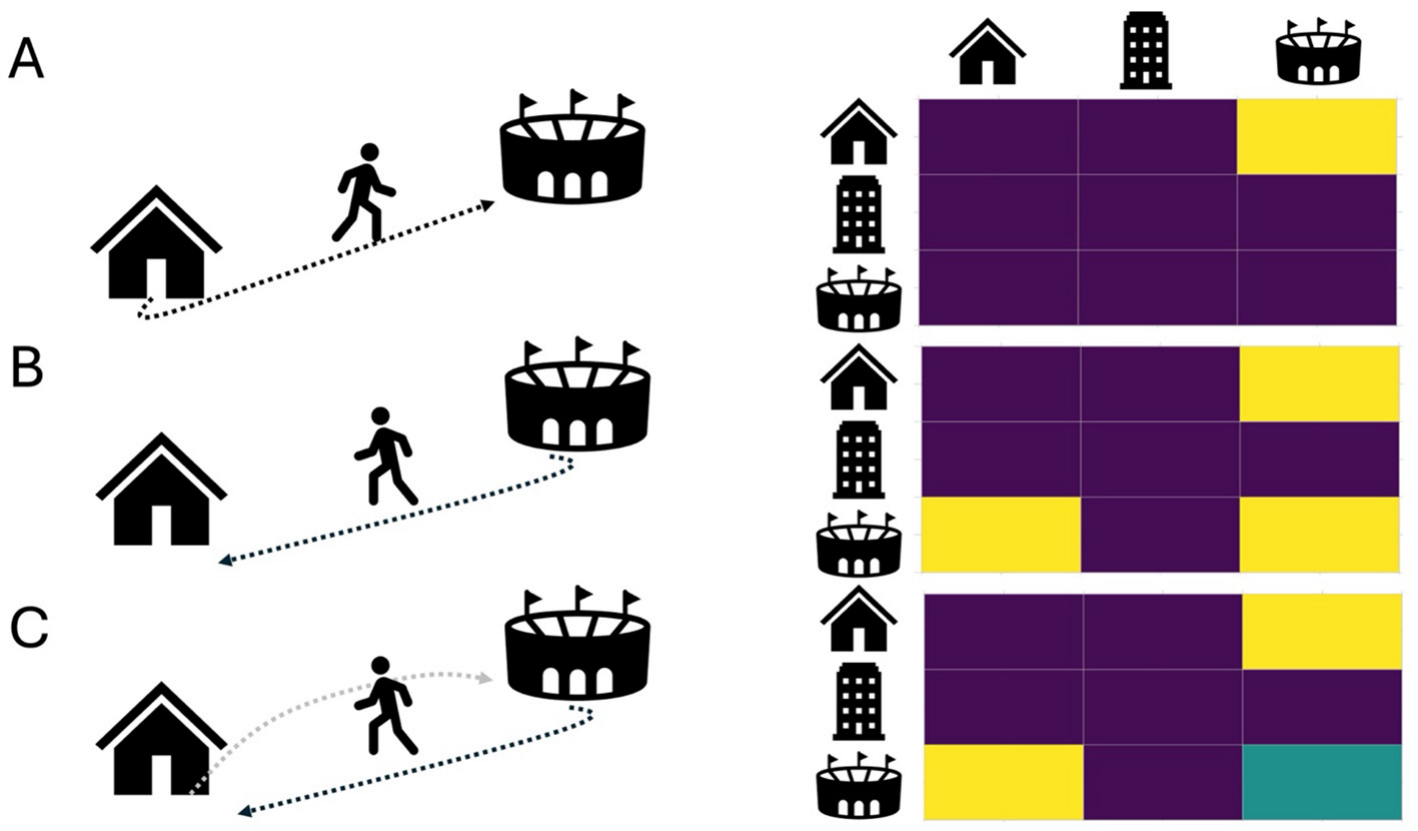
Successor matrices capture multistep transitions. **(A)** Traveling from home to Madison Square Garden results in updating the successor matrix. The right side shows a successor matrix after updating. It reflects successor probability (brighter colors = higher likelihood). Specifically, each entry is the probability of transitioning to column *j* given row *i*. Thus, because the only transition has been from home to Madison Square Garden, the successor matrix assumes that if the traveler is at home, they will go to Madison Square Garden. **(B)** Traveling from Madison Square Garen to home results in another update; however, this time, the vector representing Madison Square Garden successors assigns equal likelihood to home and Madison Square Garden. This is because the matrix already assumes that the traveler will transition from home to Madison Square Garden, and thus, being at Madison Square Garden means that they will return to Madison Square Garden in two steps. Thus, the successor matrix captures multistep transitions. **(C)** This shows the same successor matrix as shown in this **(B)**, but when the temporal discounting rate is set to 0.5, resulting in a reduced update to successor states that are multiple steps into the future.

**Figure 5:**
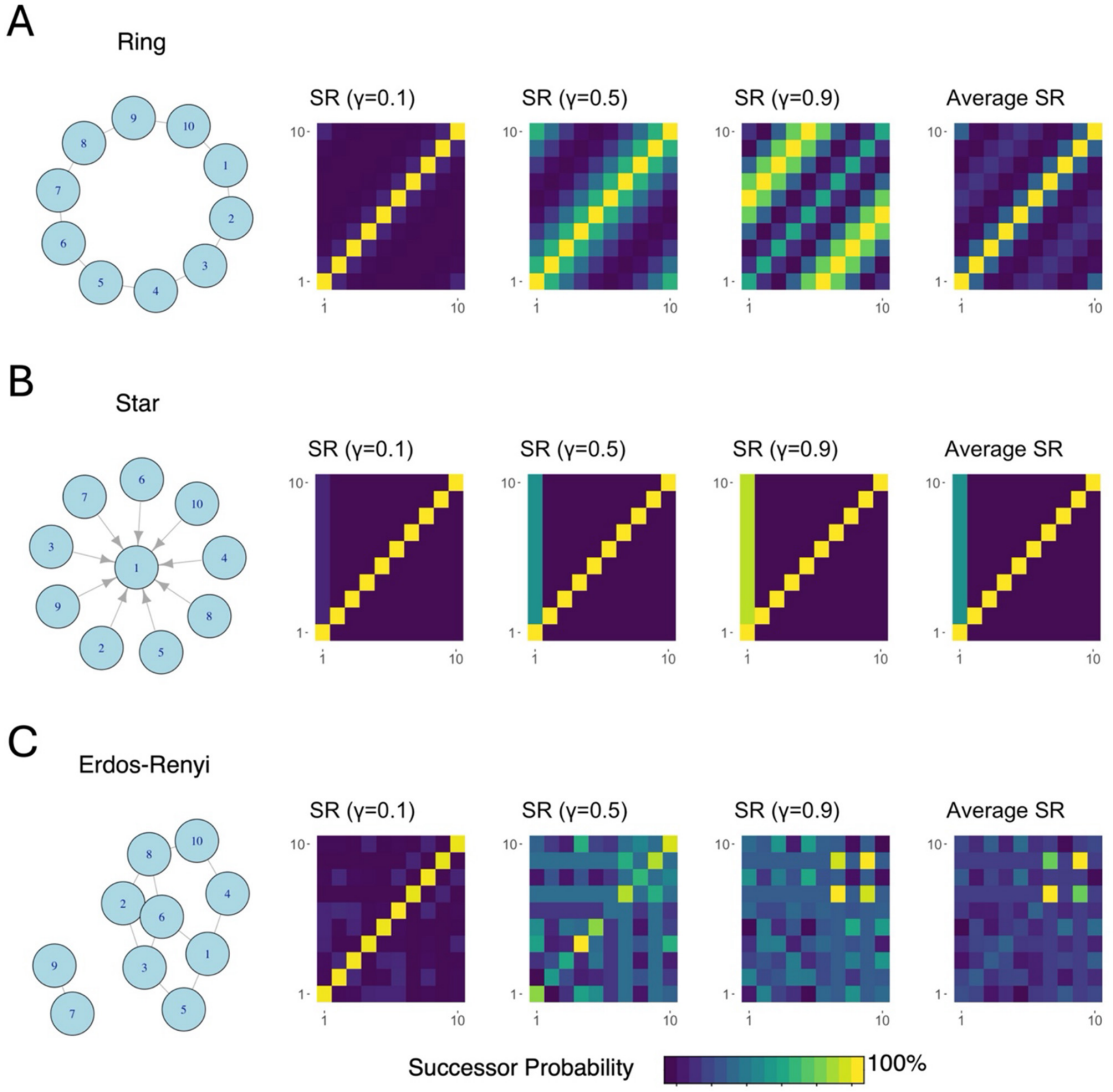
Successor representations (SRs) capture graph topographies. **(A)** This ring graph represents an environment where each state leads to two other possible states with equal likelihood. With almost no discounting (γ = 0.1), we can see that only states before and after each node (e.g., states 10 and 2 given state 1) have non-zero likelihood of transitioning to. As temporal discounting increases, multistep transitions are taken into consideration. **(B)** This graph is directed, such that every node besides 1 leads to 1. This is easy to see in the successor matrices because, regardless of temporal discounting rate, each matrix shows that every starting point (every row index) leads to node 1, or the first column. **(C)** This randomly generated graph structure is also captured by the successor representation. Notably, nodes 7 and 9 are only connected to each other, and thus, the likelihood of transitioning from 7 to 9 and from 9 to 7 is much higher than other transitions, which is captured in the yellow squares in the successor matrices.

In the last section, I neglected to talk about the temporal discounting parameter γ in the SR model. In many behavioral experiments, it is estimated as a free parameter (e.g., Son et al., 2023) to index the amount of generalization over time. How does γ index generalization? To return to the earlier example, which is also visualized in Figure 4, after the show at Madison Square Garden, say you return home. Now *s*_*t*_ is Madison Square Garden and *s*′ is home, making $I\left[s_{t}=s^{\prime }\right]+M\left(s^{\prime }\right)-M\left(s_{t}\right)=\left[1,0,0\right]+\left[0,0,1\right]-\left[0,0,0\right]=\left[1,0,1\right]$. When we add the resulting vector to the “Madison Square Garden vector,” we get [1, 0, 1]. Something interesting happened here. When we first transitioned from home to Madison Square Garden, the SR's home vector became [0, 0, 1], which is intuitive because the 1 marks the likelihood of transitioning to Madison Square Garden. But now after transitioning back home, the Madison Square Garden vector suggests that staying at Madison Square Garden is just as likely as going home, even though you never actually stayed at Madison Square Garden. This is because we never applied γ, which effectively treated γ as equal to 1. γ is bounded between 0 and 1, and the larger it is, the larger its temporal horizon; that is, large γ values update successor states that are multiple steps into the future. In the running example, you went from Madison Square Garden to home. The SR already predicts that, when at home, you will transition to Madison Square Garden. Therefore, because being at Madison Square Garden leads to returning to Madison Square Garden (in two steps), the SR assumes there is a non-zero chance of simply staying at Madison Square Garden. If γ = 0.5, the Madison Square Garden vector would be [1, 0, 0.5], meaning that multistep predictions are weighted less. This can be interpreted as generalization because two states that end up at the same endpoint will be represented similarly according to the SR. See Figure 5 for how the SR captures cognitive graphs as well.

An option's value is discounted in the SR according to how many steps away it is in the predictive map, according to γ^*r*^, where *r* is the number of steps, distance, or time points. Because the SR multiplies states by the discount rate at each step, discounting is exponential, and the infinite series can be expressed as


(13) $$\begin{array}{l} M\left(s_{t},s^{\prime }\right)=\overset{\infty }{\underset{t=0}{\sum }}\gamma^{r}\underset{s_{t}}{\sum }p\left(s^{\prime }|s_{t}\right)I\left[s_{t}=s^{\prime }\right]. \end{array}$$

To obtain estimates for the entire transition matrix *T*, we can express the SR as


(14) $$\begin{array}{l} M=\overset{\infty }{\underset{t=0}{\sum }}\gamma^{r}T^{r}, \end{array}$$

Or, using the matrix inverse,


(15) $$\begin{array}{l} M={\left(I-\gamma T\right)}^{-1}, \end{array}$$

where *I* is the identity matrix.

The discount rate in the SR can alternatively be expressed as −ln(γ) = *k*, making


(16) $$\begin{array}{l} y^{r}=exp\left(-k\cdot r\right). \end{array}$$

Equation 16 is the traditional exponential temporal discounting model using distance instead of temporal delays. Notably, Equation 16 indicates that for any SR discount rate γ∈[0, 1], there is an equivalent discount rate *k* such that the SR's discounting function can be expressed as Samuelson's exponential discounting function. As a result, choosing where to travel to next in one's *n*-dimensional decision environment can be treated as an intertemporal choice between states in a predictive map, and topological and geometric representations are implicit in the representation of discounted successor states.

Together, it is indeed plausible that we acquire spatial representations from the cognitive processing of time or, more precisely, of sequences, and these predictive maps that can recapitulate physical or abstract spaces are supported by hippocampal activity.

The idea that the same multiscale temporal discounting model can explain predictive maps is mathematically concrete, but does empirical evidence support it? No studies have explicitly tested the connection between spatial and temporal representations using the multiscale temporal discounting model; however, some nascent results hint at its biological plausibility. A particularly telling study tested the extent to which cognitive maps in hippocampal place cell activity can be explained using metrics of hyperbolic vs. Euclidean geometry (Zhang et al., 2023). This study found that hyperbolic geometry fits the data better and that such a representation maximizes the amount of spatial information contained in a population of place cells, making hyperbolic representations evolutionarily advantageous. Moreover, (Masuda et al. 2020) found that place cells, in addition to encoding spatial information, encoded temporal delays to larger-later rewards. These two studies, in conjunction with the literature on both episodic future thinking and the overlap between foraging and discounting (Harhena and Bornstein, 2023; Houser, 2024; Mikhael and Gershman, 2022; Sozou, 1998; Stevens and Stephens, 2010), entail that it is likely that the hippocampus represents space and time using the same computational principles. Showing that hippocampal spatial representations are inferred from sequences in time will be more challenging to demonstrate, as it requires showing that neural populations transform temporal sequences into predictive maps. Although I remain agnostic as to how this is achieved, a rather simple solution is that each cell in a population has exponential temporal receptive fields with a different decay rate. Thus, each cell would represent the exponential discounting model with a different decay rate. According to Equation 9, the inverse Laplace transform of such a population should yield hyperbolic discounting in space or time. This is precisely what has been predicted and found in the entorhinal cortex (Bright et al., 2020), where so-called temporal context cells track time at distinct rates. It would be interesting to see how many of these temporal context cells are also grid cells to see if the grid code also exhibits hyperbolic geometry.

It is likely that this computational principle of hyperbolic discounting via a population of exponential discounting is utilized in many brain regions due to its efficient nature. It seems specifically useful for representing magnitude information and, thus, may support the analog magnitude system (see Beck, 2015). A graded heterogeneity of time constants has been observed in the cerebellum (Guo et al., 2021) and medial prefrontal cortex (Cao et al., 2024). Further, cells in the retrosplenial cortex have been observed to integrate exponentially discounted past memories, such that proper weighting of individual exponential discounting rates resulted in a hyperbolic history integration (Danskin et al., 2023).

## Discussion

Kant shifted the philosophical tradition of investigating the nature of reality to investigating how knowledge of the nature of reality is possible. A hallmark of Kantian philosophy is that space and time serve as *a priori* intuitions that structure our mental lives and knowledge itself. This article makes the case for reducing these two intuitions to one: time. Consequently, I argued that spatial knowledge is derived from temporal experience. This theoretical framework has wide-ranging applications in cognitive science, particularly for how animals internally represent information. I used the well-researched intertemporal choice paradigm to illustrate how temporal discounting of the future is interchangeable with spatial discounting when considering travel costs associated with exploring one's environment. This overlap was made mathematically concrete by extending Sozou's (1998) temporal discounting model to predictive maps in the hippocampus. Specifically, (Sozou 1998) proposed that hyperbolic temporal discounting emerges from an ensemble of exponential discounters because of uncertainty regarding the survival of a reward over some duration. I applied this framework to a multiscale SR (Momennejad and Howard, 2018), which has been observed along the hippocampal longitudinal axis (Brunec and Momennejad, 2022). Together, the theoretical framework outlined here is biologically plausible and supported by evolutionary history. What sort of implications does this framework have?

Insights from the ideas discussed in this article can inform a number of disparate research programs in cognitive science. First, the current framework is consistent with many ideas put forth that attempt to unite all magnitude representations in the mind and brain, such as the Weber–Fechner law (J and Fechner, 1889), a theory of magnitude (Walsh, 2003), and amodal magnitude representations (Nuerk et al., 2005). The idea is that continuous variables are mapped to a generalized magnitude system in the brain for encoding. While there do appear to be different neural substrates across modalities (Cohen Kadosh et al., 2005; Hamamouche and Cordes, 2019; Sokolowski et al., 2017), evidence supports the notion that each substrate utilizes similar computational principles for representing the information. Specifically, a basis set of exponentially tuned cells with heterogeneous magnitude discounting rates encodes multiscale magnitude representations (Bright et al., 2020; Cao et al., 2021, 2024; Danskin et al., 2023; Guo et al., 2021; Zhang et al., 2023). It is highly adaptive for the brain's default to be to encode information at multiple scales because it enables flexible behavior. For example, backpacking through Europe likely requires not only a fine-grained map to be able to walk around locally but also a coarse-grained map to board the right trains. The concept of a home requires fine-grained details (e.g., its material, interior design, location) and coarse-grained details (e.g., *a place to sleep, a place to raise children, a place you return to for comfort*). Importantly, an exponential basis set is a universal function approximator (Schaul et al., 2015), meaning it can represent a potentially infinite range of functions, or tuning curves, which could be why animals exhibit considerable diversity in discounting (e.g., hyperbolic, exponential, and quasi-hyperbolic, additive). Notably, one prediction that this article makes is that the functional form of behavior in decision-making paradigms where animals choose between magnitudes depends on uncertainty. When uncertainty is high, the weights for each basis function (assuming decision is a linear combination of weighted basis functions) will be similar, making the decision approximately an average of the basis set, which creates a hyperbolic function. When uncertainty is low, one or a few weights will dominate, and tuning will be exponential. This notion has important testable implications for several fields.

First, assuming lower uncertainty facilitates more exponential discounting, intertemporal choice studies can compare exponential and hyperbolic models when the reward distributions experienced are more or less broad (i.e., uncertain). This notion also suggests that there could be order effects in intertemporal choice tasks, such that the more one learns about the reward distribution, the more their uncertainty decreases, leading to less temporal discounting on later trials. This would be particularly intriguing, given that intertemporal choice tasks are supposed to measure the stable personality trait of impulsivity. Consistent with this notion in general are studies that impose a waiting time between the presentation of the offers and when one must make a choice. Studies found that waiting periods mitigate myopia (McClure et al., 2007), purportedly because one deliberates during this period, which in turn reduces uncertainty (Gilbert and Wilson, 2007).

Together, exploring the notion that time is the most fundamental unit of cognition seems reasonable. For Bergson, every moment of time is gnawed on by the past and impregnated by the future. Every measurement of time or thought placed in spatial formatting is an abstraction from true temporal duration. Being finite and limited in the number of resources animals can employ for representation, animals must efficiently and adaptively approximate this enduring temporality. This article proposes that this is achieved in a population of cells that discounts time exponentially and whose weighted sum can give rise to hyperbolic discounting in decision-making. The computational substrates proposed here were first proposed by (Sozou 1998), who elucidated how hyperbolic discounting is rational from an ecological perspective. Sozou's (1998) model was extended to temporal difference (Kurth-Nelson and Redish, 2009) and machine (Fedus et al., 2019) learning. Both models have contributed to understanding value-based decision-making with a population-based account of temporal discounting. Specifically, (Kurth-Nelson and Redish 2009) demonstrated that a distributed set of agents with exponential discounting can approximate hyperbolic discounting. (Fedus et al. 2019) extended this model to non-hyperbolic discount functions using a neural network approach. Both models were developed to build on a novel value-based decision-making framework, but their conceptual implications have been relatively unexplored. Moreover, the neurobiological plausibility of these models is unknown. The original contribution of this article is the conceptual unification of spatial representation and temporal discounting. While the framework proposed here does not contradict past models of heterogeneous exponential discounting, it deals with the problem at a complementary level of analysis by outlining the computational task to be solved rather than the algorithmic implementation. Furthermore, the ideas presented in the current article are well grounded in neurobiology.

Intriguingly, this theoretical framework for cognitive representation has broader philosophical implications for integrating Kantian philosophy with cognitive neuroscience. First, to reiterate Bergson's stance, time is a continuous flow, meaning that, while things change, there is never a sharp discretization between events in the real world. Rather, things seamlessly overlap. Our own cells are constantly being replaced by newer cells, but our concept of self is an unbroken stream enduring through time. Consciousness, however, like science, is overwhelmed with this heterogeneity of enduring events and must discretize them to make sense of them. As a solution, consciousness, like science, spatializes time (e.g., the SR). Both artificially segment events into temporal units that can be strung together in a spatial domain like beads on a string, enabling mental (and/or mathematical) manipulation of information. This entails that space is not an *a priori* intuition, as Kant would have it, but rather a construction from *a priori* temporal intuition. Bertrand Russell was a famous empiricist who argued against Kant's idea of *a priori* intuitions (and more broadly his *transcendental idealism*), claiming instead that space and time are objective properties in the world that can be deduced from analysis and logic. While the framework put forth here is largely empirical, it is also meant to encapsulate the spatialization of time and illustrate that even time is represented spatially in the mind, brain, and science. Time itself endures and therefore cannot be known empirically—it can only be lived.

Limitations of this conceptual framework include its computational implementation. While the computational models discussed earlier are robust within their respective fields (i.e., temporal discounting and SR models), how they might be combined in, for example, a neural network architecture to recapitulate neural dynamics is unknown. Furthermore, the philosophical implications have not been thoroughly fleshed out, especially as they relate to the current understanding of the world through the lens of physics. Future work can investigate the extent to which lived time is consistent with what is known in physics.

## Data Availability

The original contributions presented in the study are included in the article/supplementary material, further inquiries can be directed to the corresponding author.
